# Through the scope, through the years: declining trends in laparoscopic common bile duct exploration

**DOI:** 10.1007/s00464-026-12963-7

**Published:** 2026-06-12

**Authors:** Matthew B. Bloom, Nicholas Gonsalves, Yufei Chen, Joshua Tseng, Sayeh Mirshojae, Rushil Rawal, Farin Amersi, Edward Phillips

**Affiliations:** Jim and Eleanor Randall Department of Surgery, Cedars-Sinai Medical Center, 8700 Beverly Blvd, Los Angeles, CA 90048 USA

**Keywords:** Laparoscopic common bile duct exploration, Choledocholithiasis, Intraoperative cholangiography, Endoscopic retrograde cholangiopancreatography, Residency training

## Abstract

**Background:**

Laparoscopic common bile duct exploration (LCBDE) remains an effective single-stage strategy for choledocholithiasis, but declining utilization has raised concerns about diminishing operative experience and training exposure among contemporary surgeons. We evaluated institutional trends in LCBDE, intraoperative cholangiography (IOC), and resident participation and identified predictors of successful duct clearance.

**Methods:**

This retrospective single-center cohort study (2012–2021) included adults undergoing laparoscopic or robotic cholecystectomy. Patients were grouped as single-stage LCBDE with cholecystectomy or two-stage management with ERCP and endoscopic sphincterotomy followed by cholecystectomy. Cases were identified using CPT and ICD procedure codes cross-referenced with operating room logs. Data were abstracted from the electronic medical record. Primary outcomes were temporal trends in LCBDE, IOC utilization, and resident participation. Secondary outcomes were predictors of successful LCBDE.

**Results:**

LCBDE succeeded in 240/291 cases (82.4%). Failures occurred exclusively during transcystic exploration, most commonly due to an inability to cannulate the cystic duct (43%). Median length of stay was shorter after successful LCBDE (1 vs 3 days, *p* < 0.001). Surgeons in successful cases had greater experience (median 17.5 vs 12.9 years, *p* = 0.03). On multivariable analysis, surgeon experience independently predicted success (OR 1.04 per year, 95% CI 1.004–1.078), whereas surgeon specialty was not significant after adjustment. Of 303 LCBDE patients, 291 met inclusion criteria; among 339 two-stage patients, 257 met inclusion criteria. Institutionally, annual LCBDE volume declined (trend *p* < 0.05), IOC utilization decreased (66% in 2018 to 54% in 2021), and resident involvement fell (91.8% in 2014 to 69.6% in 2021), with per-resident exposure declining from 2.2 to 0.9 cases per year.

**Conclusions:**

LCBDE achieves high duct-clearance rates and shorter hospitalization when successful. Surgeon experience, rather than specialty, independently predicts success, underscoring a clinically meaningful learning curve. Declines in LCBDE volume, IOC utilization, and resident exposure highlight the need for training strategies including simulation, standardized workflows, and improved case access to preserve competency.

**Graphical abstract:**

**Figure:**
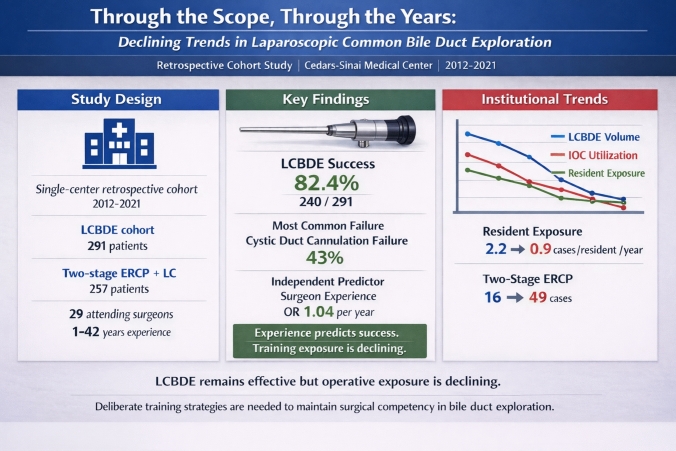

Laparoscopic common bile duct exploration (LCBDE) is a fundamental biliary procedure within general surgery training. It is most commonly performed via a transcystic approach and less frequently through choledochotomy to clear stones or debris identified on intraoperative cholangiography (IOC). When performed concurrently with laparoscopic cholecystectomy (LC), LCBDE is associated with lower morbidity, shorter length of stay (LOS), faster recovery, and superior cost-effectiveness compared with the traditional two-stage approach of LC followed by endoscopic retrograde cholangiopancreatography with sphincterotomy (ERCP/ES) [1]. These advantages are supported by additional series demonstrating high duct-clearance rates with reduced morbidity and shorter length of stay for single-stage LCBDE [2, 3]. Although open common duct exploration is now uncommon, it remains indicated in select situations.

The success of single-stage LCBDE during LC has led to the development of numerous operative techniques for duct clearance, including basket extraction, choledochoscopy, ampullary balloon dilation, antegrade sphincterotomy with lithotripsy, lavage, cystic duct dilation, and “trolling” maneuvers with baskets or balloon catheters [4, 5]. Despite these well-described advantages and technical advances, the overall volume of LCBDE has declined over time, likely reflecting increasing reliance on ERCP for the management of choledocholithiasis [6] as well as evolving operative practice patterns, including increasing adoption of robotic cholecystectomy.

This study aimed to evaluate institutional trends in LCBDE case volume during laparoscopic cholecystectomy, utilization of intraoperative cholangiography, and the frequency of resident participation. A secondary objective was to identify predictors of successful LCBDE among patients undergoing concurrent cholecystectomy for choledocholithiasis.

## Materials and methods

This retrospective, single-center cohort included adults undergoing laparoscopic or robotic cholecystectomy at a large academic teaching hospital between 2012 and 2021. During the study period, 303 patients had single-stage LCBDE performed at the time of LC, and 339 underwent two-stage management with LC and ERCP with sphincterotomy (ERCP/ES). Inclusion criteria were age ≥ 18 years and cholecystectomy (laparoscopic or robotic) performed at our institution within the study window. Exclusion criteria comprised nonbiliary indications (e.g., pancreatic malignancy or Whipple procedures), operations performed at outside hospitals, and prior cholecystostomy tube placement. These criteria were selected to eliminate unrelated pathology and to confine the analysis to cases with complete, in-house clinical decision-making and data.

Case identification utilized relevant CPT and ICD-9/10 procedure codes from a billing database. Operating room logs were cross-referenced to confirm inclusion. ICD-9 procedure and ICD-10-PCS codes were used to identify CBDE, ERCP, and sphincterotomy procedures.

Collected variables included patient demographics, surgeon experience, resident involvement, preoperative diagnosis, operative technique, and postoperative outcomes. Surgeon experience was defined as years of independent practice, obtained from institutional online profiles. Attending surgeons were categorized as Acute Care/Trauma (ACS) or MIS + General. Resident involvement and trainee level (junior resident, senior resident, fellow) were recorded based on the operative record. The dataset did not allow differentiation between levels of participation such as observation, assistance, or performance of operative steps under supervision.

During the study period, laparoscopic common bile duct exploration at our institution was most commonly performed using a transcystic approach following intraoperative cholangiography confirming choledocholithiasis. After confirmation of ductal stones, transcystic exploration was typically initiated using a wire-guided basket for stone capture. When cystic duct anatomy limited access, balloon dilation of the cystic duct–common bile duct junction was performed to facilitate passage of instruments. Stones were then extracted using a basket device, and duct clearance was confirmed with a completion cholangiogram demonstrating resolution of filling defects and free flow of contrast into the duodenum. While minor variations in instrumentation occurred between surgeons, the transcystic-first strategy and overall procedural sequence remained consistent during the study period.

The management of suspected choledocholithiasis varied depending on the referral pathway and surgeon preference. Patients presenting to surgical services commonly underwent laparoscopic cholecystectomy with intraoperative cholangiography, with laparoscopic common bile duct exploration attempted when ductal stones were identified and transcystic access was considered feasible. Alternatively, patients referred through medical or gastroenterology services often underwent ERCP with endoscopic sphincterotomy prior to surgical referral.

The primary outcomes were trends in LCBDE case volume, use of intraoperative cholangiography, and resident participation. LCBDE success was defined as documented clearance of the common bile duct. Continuous variables were summarized as mean (SD) or median (IQR) and compared using Student’s t-test or Mann–Whitney U, as appropriate; categorical variables were compared using Chi-Square or Fisher’s exact tests. Candidate predictors were screened in univariate logistic regression; variables with *p* < 0.10 were entered into the multivariable logistic regression. Statistical significance was defined as two-tailed *p* < 0.05.

## Results

### Cohort assembly

Of 303 patients who underwent LCBDE during the study period, 10 were excluded for prior ERCP performed at an outside institution and 2 for prior cholecystostomy tube placement performed externally, yielding 291 patients for analysis. Among the 339 patients who underwent two-stage LC and ERCP/ES, 63 were excluded for outside ERCP and 19 for prior cholecystostomy, yielding 257 patients in the two-stage cohort.

### Operative approach and institutional trends

Twenty-nine attending surgeons performed LCBDE during the study period, with surgeon experience ranging from 1 to 42 years in practice at the time of the operation. Among the 291 included LCBDE cases, 285 (98%) were performed transcystically and 6 (2%) via choledochotomy. Total annual cholecystectomy volume remained stable during the study period at approximately 710 cases/year (Table 1). Robotic cholecystectomy (RC) increased from 9 cases (1.2%) in 2016 to 73 (10.6%) in 2021 and further to 226 (22.7%) in 2022. IOC utilization decreased from 66% in 2018 to 54% in 2021. There was a significant downward trend in LCBDE volume over time (Poisson regression, year coefficient *p* < 0.05).

**Table 1:** Annual trend of operations and resident involvement

	Cholecystectomy			LCBDE (*n* = 291)		
	LC	RC	Total IOC^1^	Total LCBDE	Cases with resident	Cases/resident/year
Year	No. (%)			No. (%)		
2012	736 (100)	0 (0)	393 (53.4)	33 (4.5)	26 (78.8)	1.9
2013	743 (100)	0 (0)	424 (57.1)	27 (3.6)	26 (96.2)	1.3
2014	755 (100)	0 (0)	438 (58.0)	49 (6.5)	45 (91.8)	2.2
2015	727 (100)	0 (0)	419 (57.6)	49 (6.7)	47 (95.9)	2.0
2016	758 (98.8)	9 (1.2)	461 (60.1)	36 (4.7)	29 (80.6)	1.6
2017	666 (97.1)	20 (2.9)	402 (58.6)	22 (3.3)	16 (72.7)	1.0
2018	642 (96.7)	22 (3.3)	439 (65.9)	25 (3.9)	23 (92.0)	1.2
2019	648 (94.6)	37 (5.4)	400 (59.1)	17 (2.6)	11 (64.7)	0.6
2020	612 (92.2)	52 (7.8)	330 (50.1)	10 (1.6)	6 (60.0)	0.4
2021	616 (89.4)	73 (10.6)	367 (54.4)	23 (3.7)	16 (69.6)	0.9

*LC* laparoscopic cholecystectomy, *RC* robotic cholecystectomy, *IOC* intraoperative cholangiogram, *LCBDE* laparoscopic common bile duct exploration

^1^Includes robotic and laparoscopic IOC

### Resident participation

**Fig. 1:**
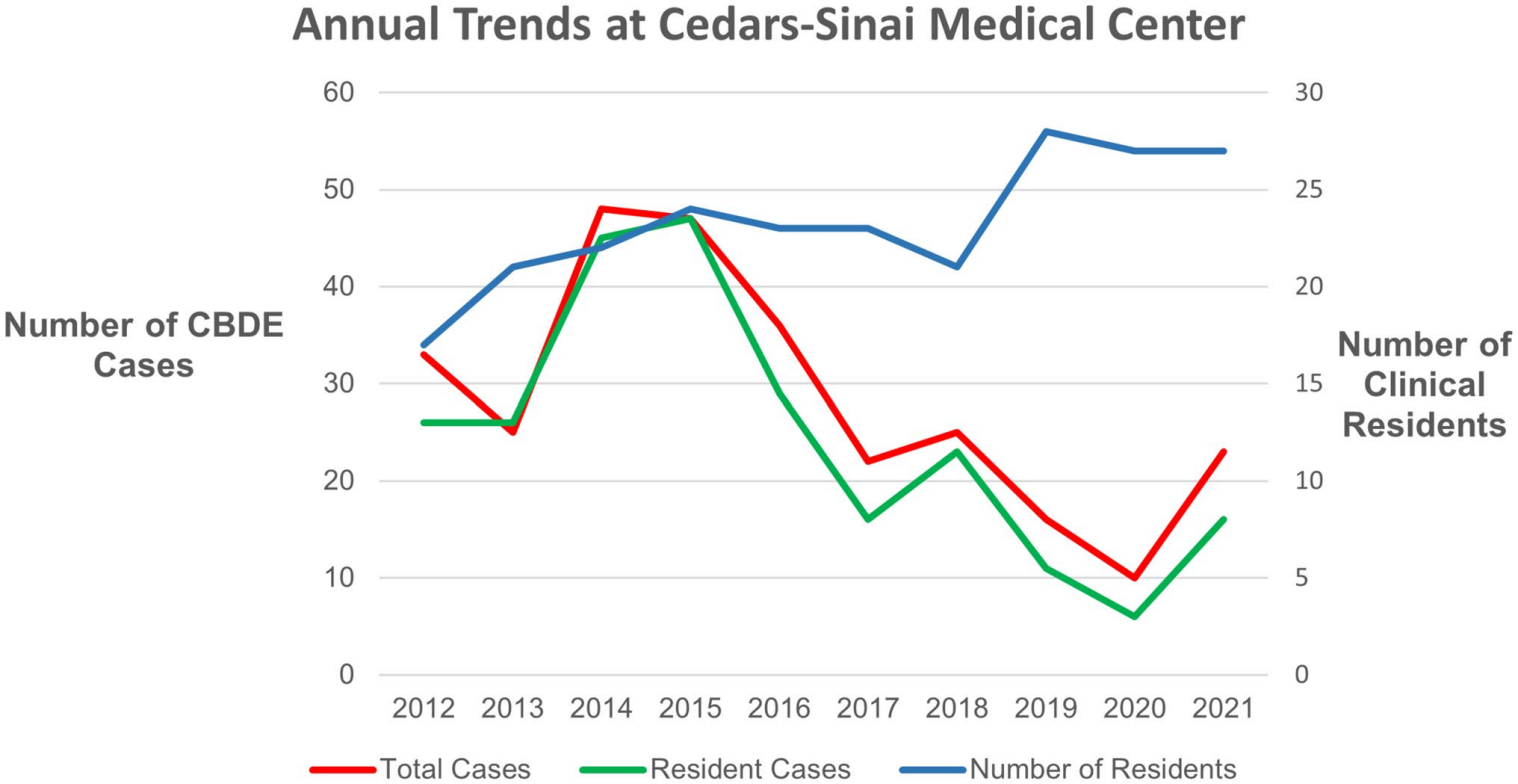
Annual trend of the number of common bile duct explorations as total cases (red) and general surgery resident-involved cases (green). The annual cohort of general surgery clinical residents is indicated by the blue line (Color figure online)

Resident-involved LCBDEs decreased from 45 (91.8%) in 2014 to 16 (69.6%) in 2021, corresponding to a drop in average per-resident case volume from 2.2 to 0.9 annually (Cochran–Armitage *p* < 0.05). Figure 1 displays yearly trends in total LCBDEs (red), resident-involved cases (green), and the number of clinical residents in the program (blue), which reflects the total number of surgical trainees annually. There were 46 cases performed without a resident. In univariate analysis using “no resident present” as the reference, junior, senior, and fellow participation were not associated with differences in success (all *p* > 0.15).

### LCBDE success, failure modes, and length of stay

LCBDE was successful in 240 of 291 cases (82.4%). Of the 51 unsuccessful cases, 45 (88.2%) underwent postoperative ERCP. No significant differences were found in patient demographics, clinical variables, or resident involvement between successful and unsuccessful cases **(**Table 2**).** Surgeons in successful cases had significantly more post-training experience (median 17.5 vs. 12.9 years; *p* = 0.03). All unsuccessful cases occurred with the transcystic approach. The most common reason for failure was inability to cannulate the cystic duct (43%). On multivariable regression, only surgeon experience was independently associated with successful LCBDE (OR 1.04; 95% CI, 1.004–1.078), Table 3.

**Table 2:** Univariate logistic regression for factors associated with successful CBDE (*n* = 291)

Predictor	Distribution/*n* (%)^1^	OR (95% CI)	*p*-value
Age at operation, years	50 (35–71)	1.0 (0.99–1.02)	0.79
Sex			
Female [ref]	197 (67.7)		
Male	94 (32.2)	0.85 (0.45–1.60)	0.62
Surgeon experience (years)	17 (10–23)	1.05 (1.02–1.08)	0.004
Surgeon specialty			
ACS/Trauma [ref]	57 (19.6)		
MIS + general	234 (80.4)	2.22 (1.12–4.38)	0.02
Trainee Level			
No resident [ref]	46 (15.8)		
Junior resident	75 (25.8)	1.45 (0.52–4.04)	0.47
Senior resident	148 (50.9)	1.55 (0.71–3.39)	0.96
Fellow	22 (7.6)	3.14 (0.63–15.6)	0.27
CBDE technique used			
Transcystic [ref]	285 (97.9)		
Choledochotomy	6 (2.1)	0.74 (0.26–2.11)	0.57

^1^ “Distribution” shows median (IQR) for continuous variables and *n* (%) for categorical variables

**Table 3:** Independent predictors of successful CBDE (Multivariable logistic regression)

Predictor	Adjusted OR (95% CI)	*p*-value
Surgeon experience (per year)	1.04 (1.004–1.078)	0.029
Surgeon specialty (MIS + general vs ACS [ref])	1.49 (0.69–3.19)	0.309

The annual volume of two-stage LC and ERCP/ES procedures increased from 16 cases in 2014 to 49 in 2021, with a transient decline in 2020 that likely reflects COVID-19-related practice changes (Table 4). In latter years, most patients underwent ERCP/ES prior to LC. Median length of stay was significantly shorter following successful LCBDE (1 vs. 3 days; *p* < 0.001).

**Table 4:** Annual trends of two-stage laparoscopic cholecystectomy and endoscopic retrograde cholangiopancreatography with endoscopic sphincterotomy

	Total	ERCP/ES then LC	LC then ERCP/ES
Year	No. No. (%) No. (%)		
2014	16	7 (43.7)	9 (56.3)
2015	20	10 (50.0)	10 (50.0)
2016	29	22 (75.8)	7 (24.2)
2017	34	16 (47.1)	18 (52.9)
2018	36	20 (55.6)	16 (44.4)
2019	43	27 (62.8)	16 (37.2)
2020	29	19 (65.6)	10 (34.4)
2021	49	33 (67.3)	16 (32.7)

*LC* laparoscopic cholecystectomy, *ERCP/ES* endoscopic retrograde cholangiopancreatography with endoscopic sphincterotomy

## Discussion

At our institution, LCBDE was performed using a largely standardized transcystic-first approach, suggesting that differences in outcomes were more likely related to surgeon experience rather than substantial variation in operative technique. Surgeon experience emerged as the sole independent predictor of successful laparoscopic common bile duct exploration (LCBDE). On multivariable analysis, each additional year in practice was associated with a 4% increase in the odds of duct clearance (adjusted OR 1.04; 95% CI 1.004–1.078), a modest but clinically meaningful effect that compounds over time. Given the multiplicative nature of odds ratios, ten additional years of experience correspond to ~ 1.48 × the odds of success (1.04^10^), highlighting the meaningful impact of accumulated clinical exposure. This is consistent with existing data showing a defined learning curve for LCBDE, with proficiency often requiring 20–30 cases [7]. In contrast, resident case volume in LCBDE has declined steadily over the study period—a trend observed both at our institution and nationally, as increasing reliance on ERCP has supplanted operative duct exploration. While MIS + General surgeons appeared more successful than ACS surgeons on univariate analysis (OR 2.22; 95% CI 1.12–4.38), this difference was no longer statistically significant after adjusting for experience (adjusted OR 1.48; 95% CI 0.69–3.19). This attenuation suggests that the observed specialty effect is largely explained by differences in time in practice, as MIS + General faculty in this cohort were older and more experienced on average. Specialty may influence case mix and team workflow, but it was not independently associated with success after adjustment for experience.

In patients managed with staged ERCP following cholecystectomy, ERCP was typically performed within the first few postoperative days. However, the need to coordinate a separate endoscopic procedure often required patients to remain hospitalized while awaiting ERCP scheduling and completion. In contrast, single-stage LCBDE allowed duct clearance during the index operation, eliminating the need for additional procedures during the same admission and contributing to the shorter hospital stay observed in this group.

During the study period, robotic cholecystectomy became increasingly common at our institution, rising from 1% of cholecystectomies in 2016 to 10.6% in 2021. Despite this increase, the vast majority of bile duct explorations in this cohort were performed laparoscopically, limiting our ability to directly compare laparoscopic and robotic LCBDE outcomes. Nevertheless, the expansion of robotic cholecystectomy may influence how surgeons approach the management of choledocholithiasis. Factors such as operating room workflow, robotic docking, and integration of fluoroscopy may affect the routine use of intraoperative cholangiography and transcystic exploration. As robotic approaches continue to expand, understanding how these factors influence bile duct exploration will be important to ensure that surgeons maintain familiarity with single-stage operative management.

This shift may partly explain the concurrent decline in IOC utilization. Our institution has historically emphasized routine intraoperative cholangiography, reflecting the longstanding influence of Dr. George Berci, a pioneer in operative biliary imaging and cholangiography. This longstanding institutional practice has supported a surgery-first approach to suspected choledocholithiasis, with laparoscopic cholecystectomy and routine operative cholangiography performed prior to consideration of ERCP in many patients. The decline in IOC use observed even within such an environment suggests that the broader reduction in operative bile duct evaluation may be even more pronounced at centers without similar institutional advocacy. These trends coincide with national signals of diminishing operative exposure to complex biliary procedures during training [8].

The reduction in LCBDE case volume is concerning from a quality and training standpoint. As CBDE becomes less common, technical complications have been reported to rise, emphasizing the need for deliberate skill maintenance and structured training pathways [9]. Contemporary survey data further indicate that most general surgeons prefer ERCP for choledocholithiasis, with only a minority regularly performing LCBDE, which likely reduces resident exposure to operative duct exploration [10]. These data, combined with our findings, suggest that several converging forces—including increasing ERCP utilization, expanding adoption of robotic cholecystectomy, and surgeon preference for endoscopic management—are eroding opportunities to teach LCBDE during general surgery training. Given that surgeon experience correlates with success, a continued decline in resident exposure may translate into reduced LCBDE effectiveness in future practice. Educational strategies that strengthen intraoperative coaching and structured feedback have been associated with improved resident autonomy and performance in complex biliary surgery, reinforcing the value of formalized training interventions [11].

Although a prior Cochrane meta-analysis lacked sufficient data to compare length of stay (LOS) between single-stage and two-stage management strategies, our data indicate that successful LCBDE is associated with significantly shorter LOS, which may confer clinical and economic advantages [12, 13]. Notably, in 2014, our patients were more likely to undergo LC prior to ERCP/ES, but this pattern has since reversed. Furthermore, patients admitted to the medical service for choledocholithiasis underwent ERCP/ES prior to LC in 75% of cases over the study period. In response, our institution implemented a policy in January 2023 designating general surgery as the admitting service for patients presenting to the emergency department with choledocholithiasis. We hypothesize that this change will increase surgical management of biliary stones and reduce hospital LOS. This finding is aligned with prior comparative analyses reporting superior outcomes and shorter LOS with LCBDE compared to ERCP followed by cholecystectomy [1–3].

The decline in per-resident LCBDE exposure, despite rising trainee numbers, likely reflects a combination of reduced institutional case volume, increasing reliance on ERCP, and logistical challenges in coordinating resident availability with index cases. The ongoing decline in LCBDE likely carries important clinical consequences not only for patients, but also for surgeons who must occasionally perform open bile duct exploration in complex scenarios. Open CBDE is now infrequent and typically undertaken after failed endoscopic attempts or in patients with unfavorable or altered anatomy (e.g., after Roux-en-Y gastric bypass). In these settings, the technical principles and psychomotor skills honed during LCBDE, including precise ductal mapping and cholangiographic interpretation, wire and basket manipulation, selective ampullary balloon dilation, choledochoscopy-guided clearance, and decision-making around choledochotomy and closure, are directly transferable to the open operation. As LCBDE exposure wanes, so too does routine familiarity with these techniques, potentially widening the gap between what is required in rare, high-stakes open explorations and the skills available in contemporary practice. Preserving LCBDE within training pathways, therefore, is not only about maintaining a minimally invasive option; it is also a pragmatic strategy to safeguard competence in open CBDE when endoscopic therapy fails or anatomy precludes it.

This study has several limitations. First, its retrospective, single-center design may limit generalizability to other institutions with different operative volumes, training structures, or ERCP availability. Second, procedural success was defined by documentation of ductal clearance rather than objective imaging or long-term outcomes, which may introduce bias. Third, surgeon experience was estimated by years in practice rather than specific LCBDE case volume, which may not fully reflect technical proficiency. Additionally, although resident involvement was recorded, the extent of hands-on participation could not be quantified. Finally, selection bias is possible, as more complex or high-risk cases may have been preferentially managed by ERCP, potentially underestimating the success rate of LCBDE in more challenging scenarios.

In summary, resident experience with LCBDE continues to decline, raising concerns about the preparedness of future surgeons to perform this complex but effective procedure. To produce high-value, cost-conscious surgeons, residency programs should ensure that trainees achieve competency in interpreting IOC and performing LCBDE prior to graduation. This may be accomplished through simulation-based training in laparoscopic and robotic techniques, optimizing resident access to relevant cases, and encouraging routine use of IOC in both LC and RC. Several simulation-based models have been developed to teach laparoscopic bile duct exploration outside the operating room, including low-cost laparoscopic box trainers incorporating synthetic or ex vivo bile duct models. These platforms allow trainees to practice wire manipulation, basket extraction, and transcystic exploration techniques in a controlled environment and may help mitigate declining operative exposure. Simulation-based training has shown strong efficacy in improving resident performance and confidence in LCBDE, particularly through structured curricula and low-cost validated models [14–16]. Maintaining proficiency in ductal exploration will require deliberate investment in resident education to ensure technical skill, clinical judgment, and procedural confidence persist beyond training. While these findings are limited to a single academic center, the observed trends likely reflect broader national shifts and merit further multi-institutional study.

## References

[1] Koc B, Karahan S, Adas G, Tutal F, Guven H, Ozsoy A (2013) Comparison of laparoscopic common bile duct exploration and endoscopic retrograde cholangiopancreatography plus laparoscopic cholecystectomy for choledocholithiasis: a prospective randomized study. Am J Surg 206(4):457–463 23871320 10.1016/j.amjsurg.2013.02.004

[2] Pan L, Chen M, Ji F, Yang K (2021) Outcomes of laparoscopic common bile duct exploration: a meta-analysis. Surg Endosc 35(7):3130–3141. 10.1007/s00464-020-07994-w

[3] Chouillard EK, Sui Z, Ngo P et al (2022) Bile duct clearance: single-stage surgery versus two-stage ERCP and cholecystectomy—a matched cohort study. HPB (Oxford) 24(1):67–73. 10.1016/j.hpb.2021.09.006

[4] Lyass S, Phillips EH (2006) Laparoscopic transcystic duct common bile duct exploration. Surg Endosc 20(Suppl):S441–S445 16544067 10.1007/s00464-006-0029-0

[5] Suwatthanarak T, Akaraviputh T, Phalanusitthepha C et al (2021) Outcomes of laparoscopic common bile duct exploration by chopstick technique in choledocholithiasis. JSLS 25(2):e2021.00008 34248338 10.4293/JSLS.2021.00008 PMC8245271

[6] Pan L, Zhang H, Zeng Y et al (2016) Nationwide trends in the management of choledocholithiasis: 1998–2013. JAMA Surg 151(12):1125–1130. 10.1001/jamasurg.2016.231227556900

[7] Jiang Y, Zhang W, Liu Y et al (2023) Learning curve of laparoscopic common bile duct exploration: a systematic review. Surg Endosc 37(12):9458–9468. 10.1007/s00464-023-10326-4

[8] Melendez MM, Fulton JL, Alarcon LF et al (2008) The challenges of resident training in complex hepatobiliary procedures: ACGME case log analysis from 1994–2007. J Surg Educ 65(6):512–515. 10.1016/j.jsurg.2008.08.00819059186

[9] Livingston EH, Rege RV (2005) Technical complications are rising as common duct exploration is becoming rare. J Am Coll Surg 201(3):426–433. 10.1016/j.jamcollsurg.2005.04.029 16125077 10.1016/j.jamcollsurg.2005.04.029

[10] Baucom RB, Feurer ID, Shelton JS, Kummerow K, Holzman MD, Poulose BK (2016) Surgeons, ERCP, and laparoscopic common bile duct exploration: do we need a standard approach for common bile duct stones? Surg Endosc 30:414–423 26092008 10.1007/s00464-015-4273-z

[11] Nyren MQ, Ahmed A, Eaton D et al (2023) Formative feedback improves resident autonomy and performance in complex biliary procedures: a multicenter study. World J Emerg Surg 18:23. 10.1186/s13017-023-00490-z36966323

[12] Dasari BV, Tan CJ, Gurusamy KS et al (2013) Surgical versus endoscopic treatment of bile duct stones. Cochrane Database Syst Rev 9:CD003327 10.1002/14651858.CD003327.pub3 23999986

[13] Tang CN, Siu WT, Ha JP et al (2008) LCBDE versus ERCP in the management of common bile duct stones: a randomized trial. Surg Endosc 22(2):364–368. 10.1007/s00464-007-9498-3

[14] Sánchez A, Devitt KS, Brown CJ, Ball CG (2012) Validation of a low-cost four-task laparoscopic bile duct exploration training model. JSLS 16(4):620–625. 10.4293/108680812X13517013316990

[15] Yang AD, Lidor AO, Chang DC et al (2022) Simulation-based training curriculum for laparoscopic common bile duct exploration improves performance and autonomy. Surg Endosc 36(3):1487–1493. 10.1007/s00464-021-08527-w

[16] Hernandez JM, Brown KM, Russell GB et al (2014) Structured simulation training enables mastery of laparoscopic bile duct exploration. Surg Endosc 28(7):2051–2058. 10.1007/s00464-014-3450-2

